# Supplementary material for: Stable heritability of type 1 diabetes in a Swedish Nationwide Cohort Study
Source: Nat Commun. 2025 Jun 17;16:5327. doi: 10.1038/s41467-025-60813-2 (PMC12174315; doi:10.1038/s41467-025-60813-2)
Supplement: Supplementary file 1 — Supplementary Information [file 41467_2025_60813_MOESM1_ESM.pdf]

Stable Heritability of Type 1 Diabetes in a Swedish Nationwide Cohort Study

List of supplementary materials

Supplementary Figures

Supplementary Figure 1. The incidence of type 1 diabetes at different age subgroups by birth years

Supplementary Figure 2. The path diagram shows the moderation of birth year on A and E based on full sibling pairs

Supplementary Figure 3. Proportion of variance in type 1 diabetes explained by genetic (heritability) and non-shared environmental factors according to birth year based on other models

Supplementary Figure 4. Associations of infections with type 1 diabetes

Supplementary Figure 5. The associations of serious life events with type 1 diabetes

Supplementary Figure 6. Distribution of type 1 diabetes-related perinatal factors over birth year

Supplementary Figure 7. Flow chart of the study design

Supplementary Tables

Supplementary Table 1. Basic characteristics of participants by birth year

Supplementary Table 2. Number of concordant and discordant pairs of full siblings for type 1 diabetes overall and at age 0-6, 7-12, and 13-18 years

Supplementary Table 3 Comparisons between models with and without moderation of birth year* on different components of T1D variance based on all possible sibling pairs

Supplementary Table 4. Sensitivity analysis comparing models with and without moderation of birth year* on different components of type 1 diabetes variance based on one sibling pair from each family

Supplementary Table 5. The difference in heritability between different birth years

Supplementary Table 6. Basic characteristics and heritability of type 1 diabetes (0-18 years) in the simulated cohort as compared to the original cohort

Supplementary Table 7. Environmental factors associated with type 1 diabetes identified through the literature review

Supplementary Table 8. ICD codes for infection

Supplementary Methods

Supplementary Method 1. Assessment of childhood-onset type 1 diabetes

Supplementary Method 2. PubMed search terms to identify type 1 diabetes-related environmental factors

Supplementary Method 3. Assessment of environmental factors

Supplementary Method 4. Fitting liability threshold model with moderation effects by birth year

Supplementary Method 5. Simulation analysis to estimate expected heritability in a scenario where the increasing incidence of type 1 diabetes is completely driven by environmental factors

Supplementary Method 6. Analysis of the association between environmental factors and type 1 diabetes

Supplementary Method 7. Estimating proportion of increasing incidence of type 1 diabetes (birth year 2000 vs 1982) explained by the changing prevalence of childhood overweight/obesity

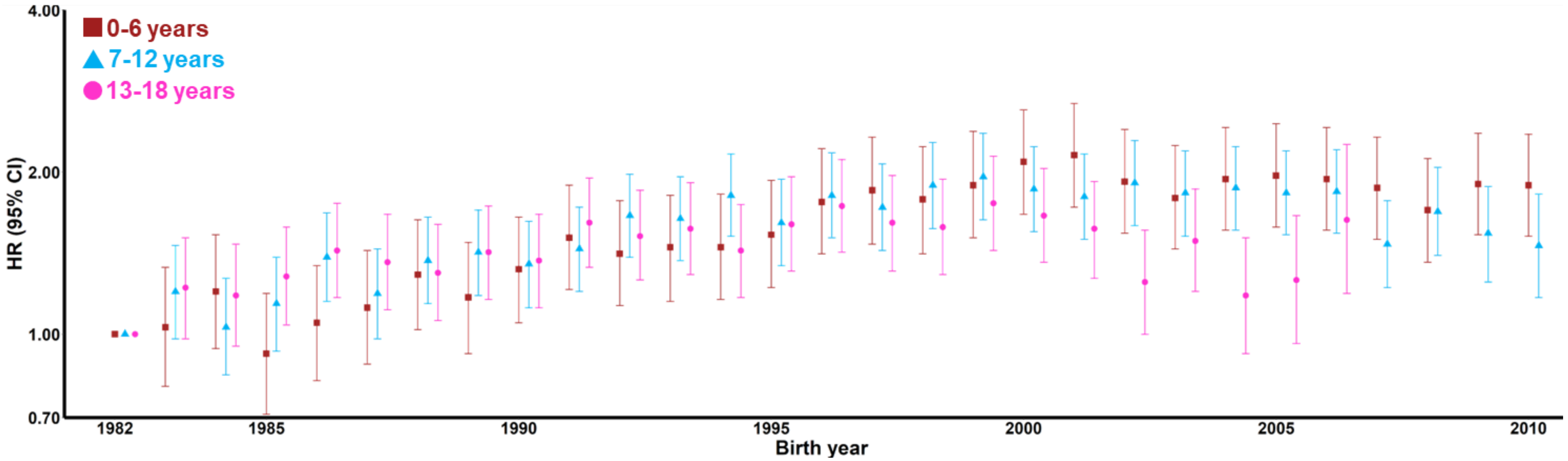

Supplementary Figure 1. The incidence of type 1 diabetes at different age subgroups by birth year

HR: hazard ratio; CI: confidence interval.

The solid squares, triangles, and circles represent the point estimates for HRs while the error bars represent the 95% CIs. HR (95% CI) of type 1 diabetes in each birth year was calculated using Cox models, with the birth year of 1982 as the reference group, with attained age as the time scale and with adjustment for sex. Cluster-robust standard errors were estimated to correct for the dependence among individuals born by the same mother. The analysis of age 0-6 years involved the full cohort of children (n=2,928,704). The analyses for the age 7-12 and 13-18 years were based on 2,875,554 and 2,466, 184 participants who were still alive and free of diabetes before age 7 and 13 years, respectively.

The time trend for ages 13-18 was unstable after birth year 2005 due to short follow-up and few events.

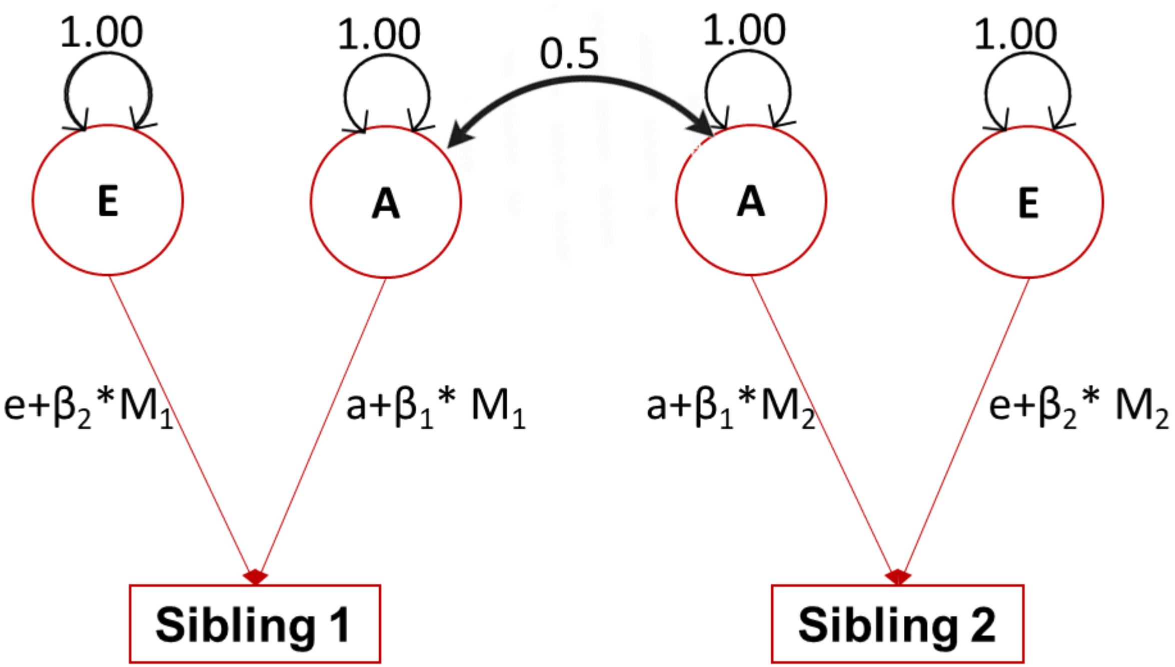

Supplementary Figure 2. The path diagram shows the moderation of birth year on A and E based on full sibling pairs

A: additive genetic component; E: non-shared environment component; β_1 and β_2 are the coefficients for moderation of birth year variable (M, calculated as (birth year-1996)/28) on the A and E component.

(A)

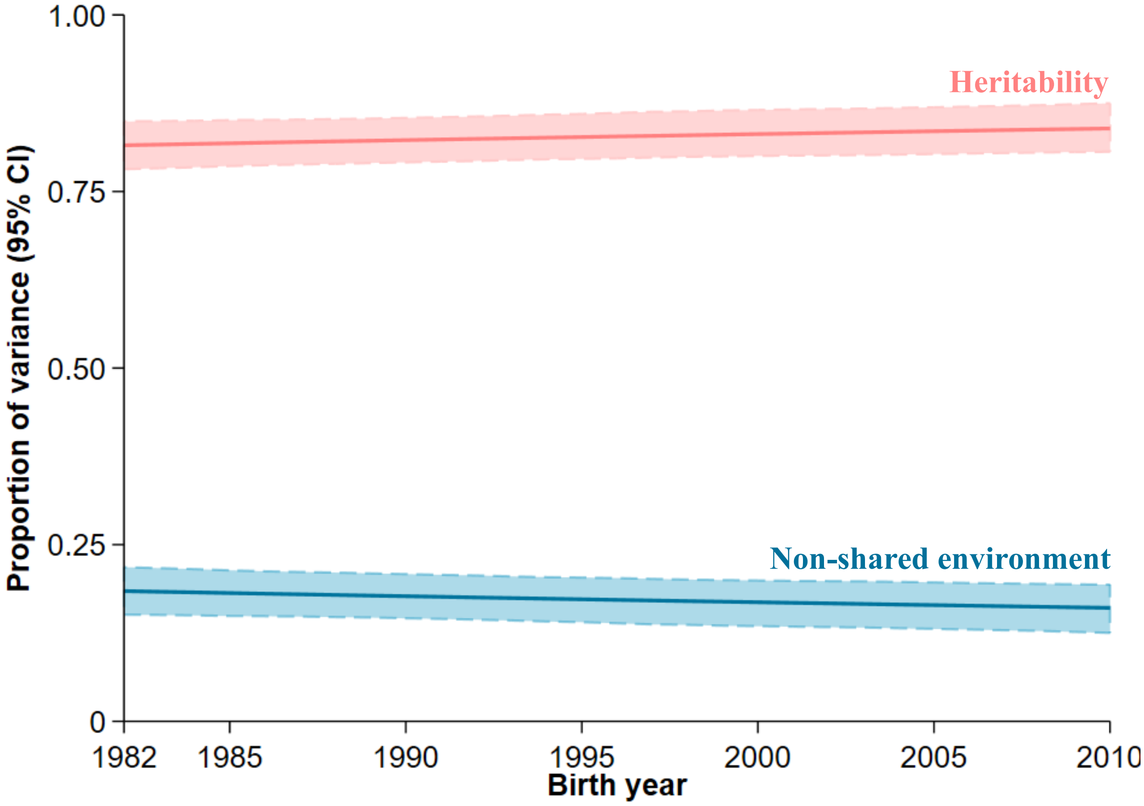
Heritability
Non-shared environment
Proportion of variance (95% CI)
1.00
0.75
0.50
0.25
0
1982
1985
1990
1995
2000
2005
2010
Birth year

(B)

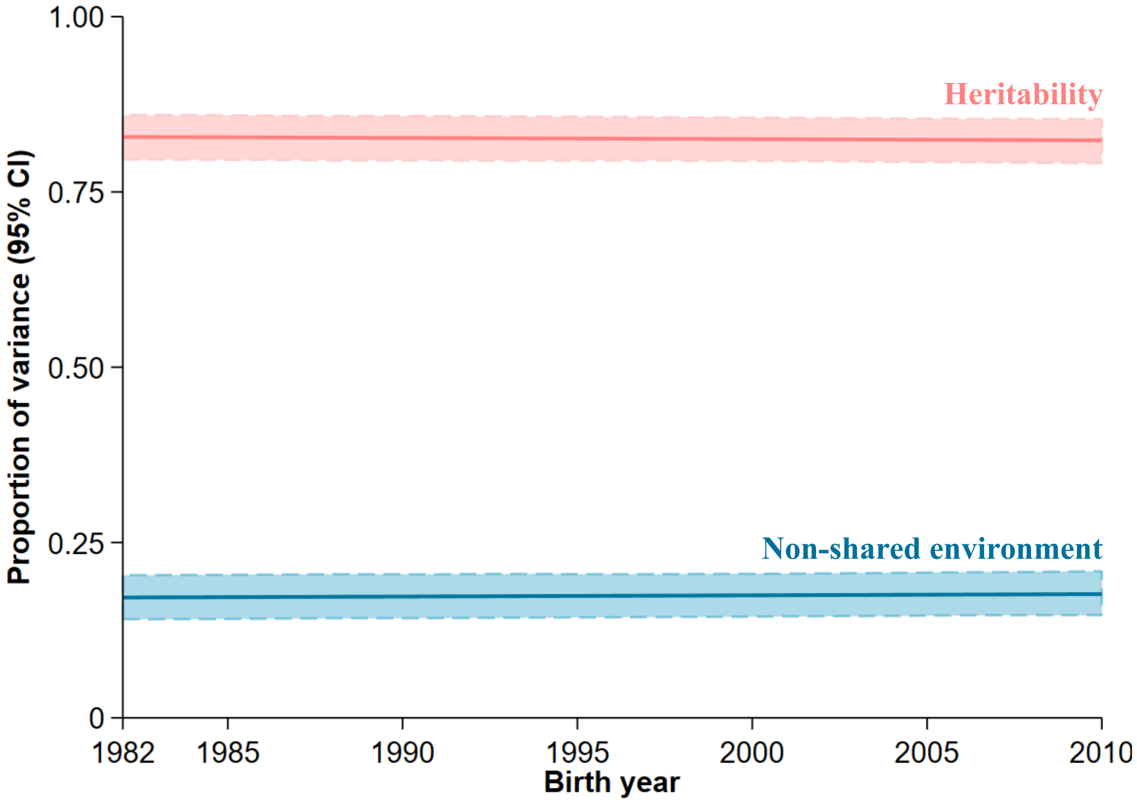
Heritability
Non-shared environment
Proportion of variance (95% CI)
1.00
0.75
0.50
0.25
0
1982
1985
1990
1995
2000
2005
2010
Birth year

(C)

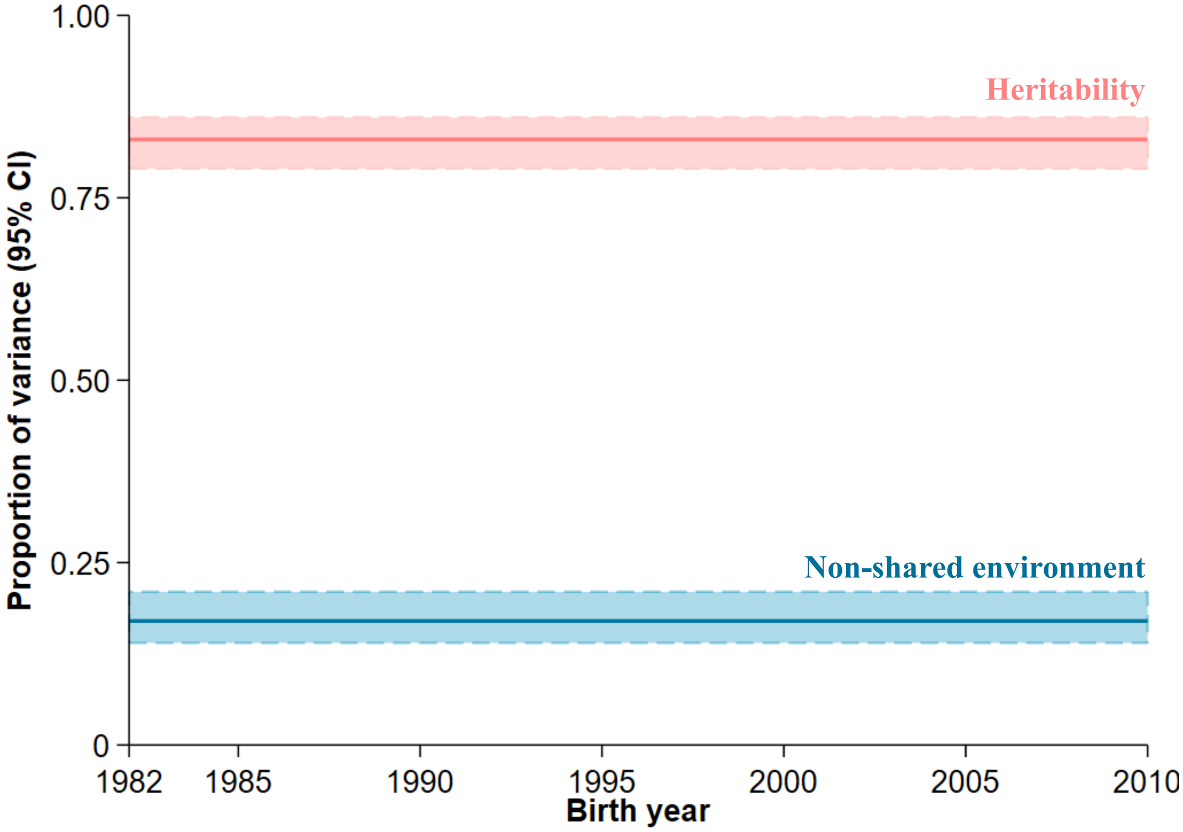

Supplementary Figure 3. Proportion of variance in type 1 diabetes explained by genetic (heritability) and non-shared environmental factors according to birth year based on other models

CI: confidence interval.

The solid pink and blue lines represent the point estimates for the proportions of type 1 diabetes variance contributed by genetic (heritability) and non-shared environment components, respectively, and the shaded areas represent 95% CIs estimated using bootstrapping approach. Analyses were based on the AE model with moderation of birth year only on the genetic component (model 2, Supplementary Figure 3A), only on the environmental component (model 3, Supplementary Figure 3B), or no moderation of birth year on any component (model 4, Supplementary Figure 3C), with adjustment for sex and birth year for the liability threshold.

Since there is no moderation of birth year on either the genetic or environmental component in model 4, the heritability estimated from model 4 is, by definition, the same (0·83, 95% CI: 0·79, 0·86) in each birth year.

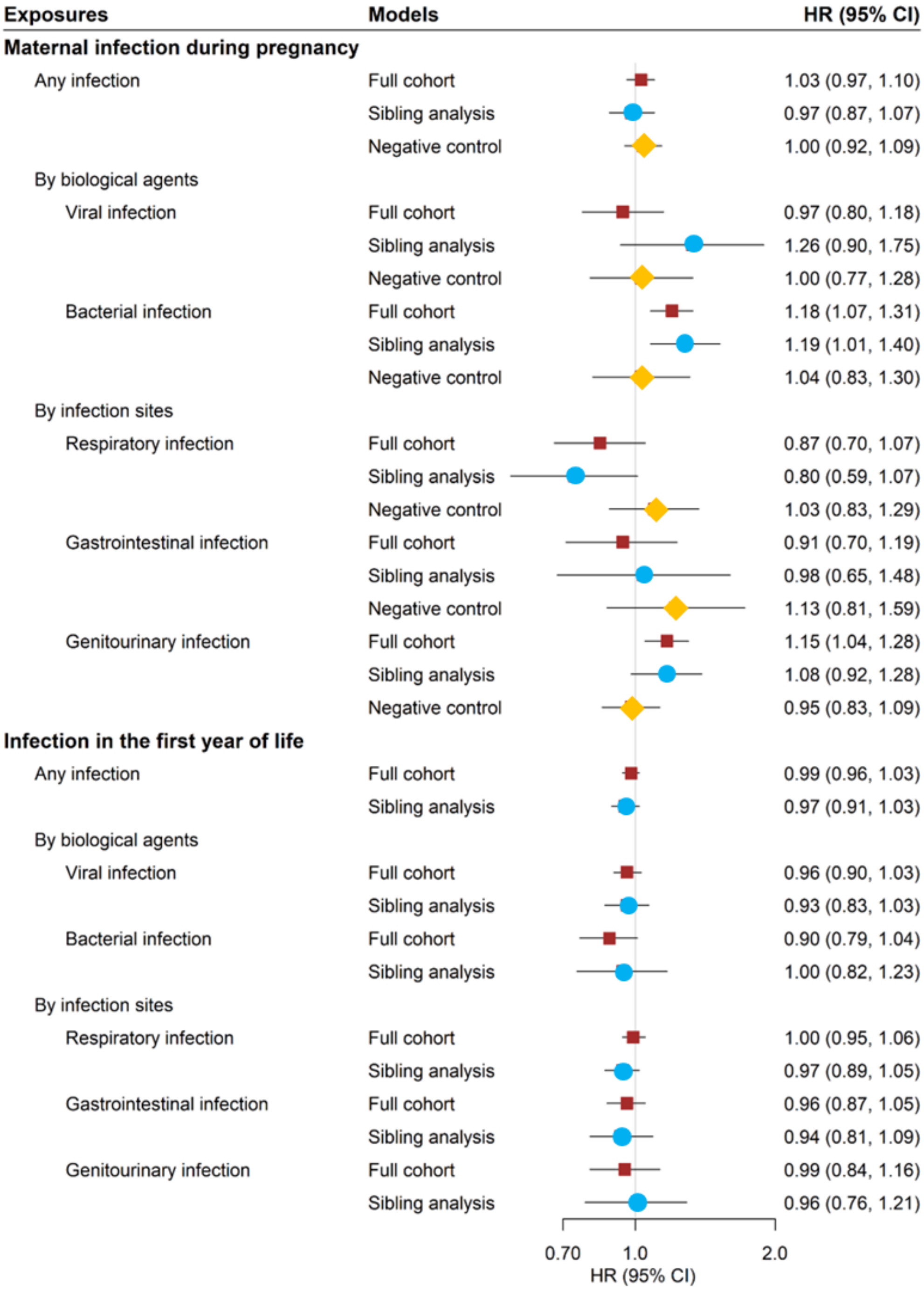

Supplementary Figure 4. Associations of infections with type 1 diabetes

HR: hazard ratio; CI: confidence interval.

The solid squares and circles represent the HRs while the error bars represent the 95% CIs. The HRs (95% CIs) of type 1 diabetes in relation to environmental infections in the full cohort (n=2,928,704) were estimated using Cox models with adjustment for birth year, sex, maternal age at delivery, maternal BMI and smoking during pregnancy, and parental country of birth and history of type 1 diabetes, with cluster-robust standard errors to account for correlation between children born by the same mother. The sibling analysis (n=41,618) was to control for unmeasured familial confounding. We also analyzed type 1 diabetes risks in relation to maternal pre-pregnancy infection (infection within 1

year prior to pregnancy) in the full cohort, as a negative control to detect potential bias in the analysis of maternal infection during pregnancy.

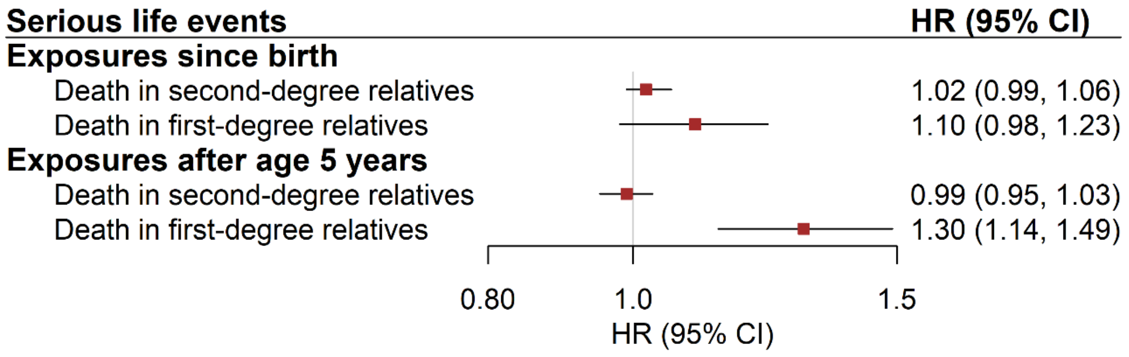

Supplementary Figure 5. The associations of serious life events with type 1 diabetes

HR: hazard ratio; CI: confidence interval.

The solid squares represent the point estimates for HRs while the error bars represent the 95% CIs. The HRs (95% CIs) of type 1 diabetes in relation to environmental infections in the full cohort were estimated using Cox models with adjustment for birth year, sex, maternal age at delivery, maternal BMI and smoking during pregnancy, and parental country of birth and history of type 1 diabetes, with cluster-robust standard errors to account for correlation between children born by the same mother. The follow-up duration was calculated from birth when the exposure was assessed since birth while the duration was calculated from age 5 years if the exposure was assessed after age 5 years.

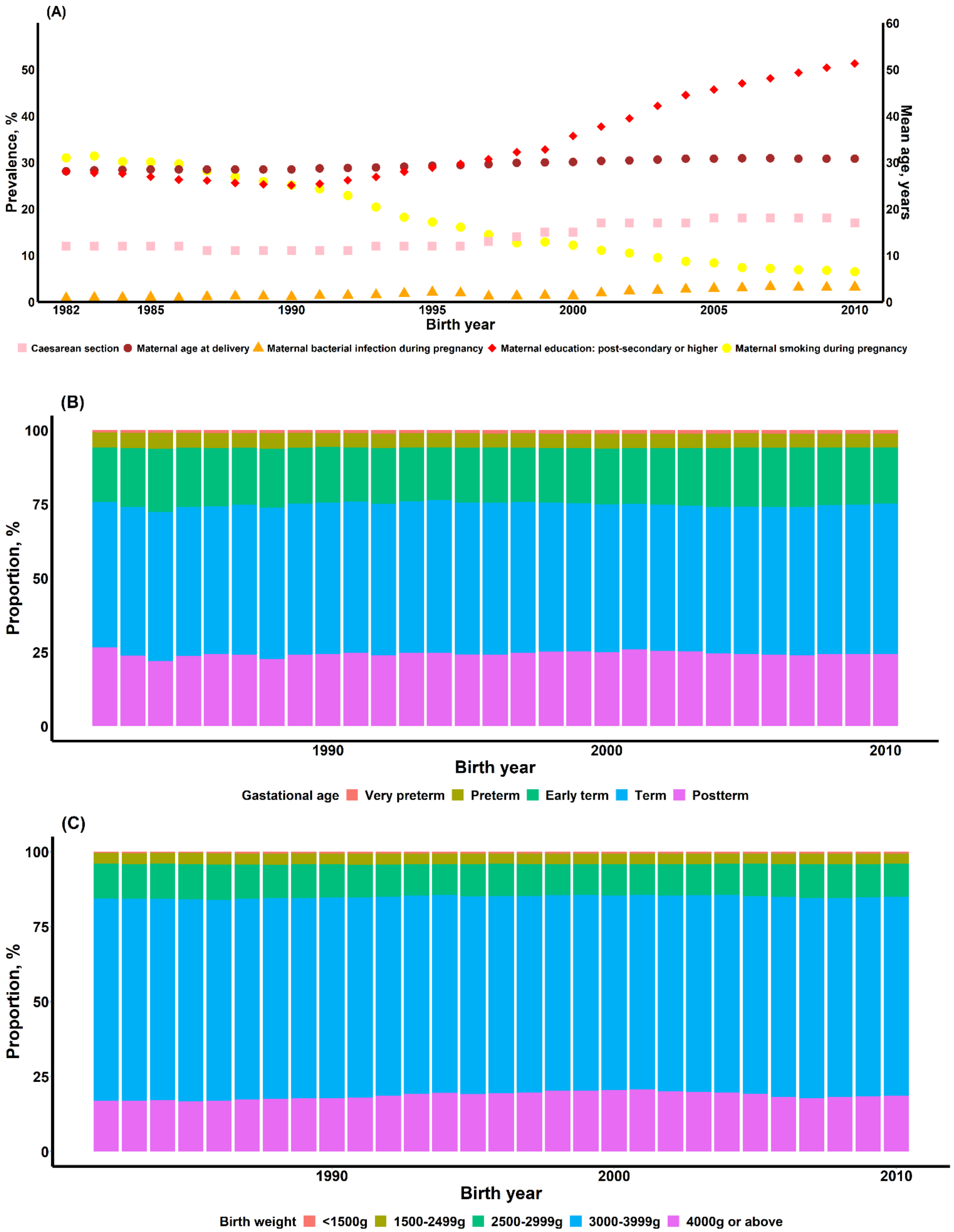

Supplementary Figure 6. Distribution of type 1 diabetes-related perinatal factors over birth year

The distribution of different factors was based on participants without missing data for the corresponding factors (n=2,928,704 for mode of delivery and maternal age at delivery, n=2,913,572 for maternal bacterial infection during pregnancy, n=2,911,977 for maternal education, n=2,717,033 for maternal smoking during pregnancy, n=2,923, 624 for gestational age, n=2,919,163 for birth weight).

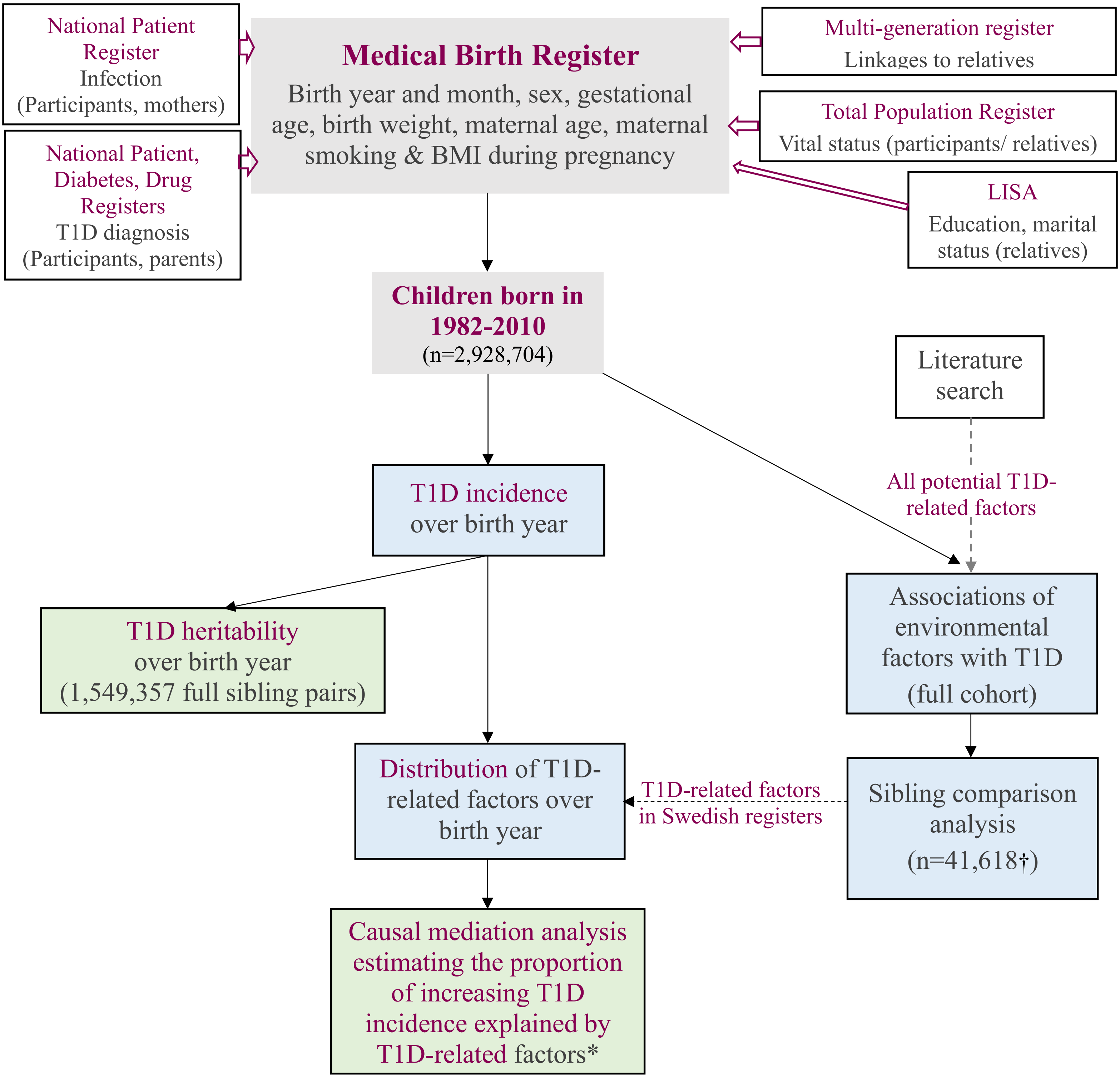

Supplementary Figure 7. Flow chart of the study design

T1D: type 1 diabetes
*Only for the birth cohort period when the incidence of T1D increased steadily. The first and last birth years during that period were used as the exposure in the causal mediation analyses.
† Only families with at least one T1D case were included since families without any T1D case were not informative for risk estimates in sibling comparison analysis.

Supplementary Table 1. Basic characteristics of participants by birth year

Basic characteristics	Birth year 1982-1990		Birth year 1991-2000		Birth year 2001-2010	
	Total	T1D	Total	T1D	Total	T1D
All	915,157	5,566	1,004,749	8,233	1,008,798	6,287
Median age at diagnosis in years	-	11·1	-	10·6	-	8·3*
Boys, n (%)	470,468 (51·4)	3,005 (54·0)	515,798 (51·3)	4,578 (55·6)	518820 (51·4)	3353 (53·3)
Maternal age in years, mean(SD)	28·4 (5·1)	28·7 (5·1)	29·3 (5·0)	29·4 (4·9)	30·7 (5·1)	30·9 (5·1)
Maternal marital status during pregnancy, n (%)						
Cohabiting with children's fathers	789,823 (86·3)	4,852 (87·2)	872,528 (86·8)	7,252 (88·1)	906757 (89·9)	5704 (90·7)
Not cohabiting with children's fathers	46,493 (5·1)	261 (4·7)	47,481 (4·7)	332 (4·0)	53260 (5·3)	295 (4·7)
Unknown	78,841 (8·6)	453 (8·1)	84,740 (8·4)	649 (7·9)	48781 (4·8)	288 (4·6)
Maternal educational level, n (%)						
Pre-secondary or lower	199,440 (21·8)	1,193 (21·4)	162,747 (16·2)	1,209 (14·7)	117657 (11·7)	636 (10·1)
Upper-secondary or high school	468,202 (51·2)	2,953 (53·1)	545,565 (54·3)	4,698 (57·1)	425811 (42·2)	2848 (45·3)
Post-secondary or higher	239,541 (26·2)	1,414 (25·4)	293,037 (29·2)	2,321 (28·2)	459977 (45·6)	2792 (44·4)
Unknown	7,974 (0·9)	6 (0·1)	3,400 (0·3)	5 (0·1)	5353 (0·5)	11 (0·2)
Paternal educational level, n (%)						
Pre-secondary or lower	238,039 (26·0)	1,434 (25·8)	183,713 (18·3)	1,424 (17·3)	124650 (12·4)	673 (10·7)
Upper-secondary or high school	431,542 (47·2)	2,685 (48·2)	533,727 (53·1)	4,604 (55·9)	491109 (48·7)	3337 (53·1)
Post-secondary or higher	230,134 (25·1)	1,391 (25·0)	278,257 (27·7)	2,178 (26·5)	380194 (37·7)	2230 (35·5)
Unknown	15,442 (1·7)	56 (1·0)	9,052 (0·9)	27 (0·3)	12845 (1·3)	47 (0·7)
Maternal BMI during pregnancy in kg/m^2, mean (SD)	22·0 (3·1)	22·2 (3·2)	23·9 (4·0)	24·3 (4·2)	24·6 (4·5)	24·9 (4·6)
Maternal smoking during pregnancy, n (%)						
No	579,906 (63·4)	3,790 (68·1)	780,542 (77·7)	6,653 (80·8)	879514 (87·2)	5548 (88·2)
Yes	229,943 (25·1)	1,204 (21·6)	168,490 (16·8)	1,155 (14·0)	78638 (7·8)	426 (6·8)
Unclear	105,308 (11·5)	572 (10·3)	55,717 (5·5)	425 (5·2)	50646 (5·0)	313 (5·0)
Maternal infection during pregnancy, n (%)						
No	887,522 (97·0)	5,390 (96·8)	969,651 (96·5)	7,928 (96·3)	912627 (90·5)	5713 (90·9)
Yes	25,417 (2·8)	165 (3·0)	32,778 (3·3)	286 (3·5)	85577 (8·5)	500 (8·0)
Unknown	2,218 (0·2)	11 (0·2)	2,320 (0·2)	19 (0·2)	10594 (1·1)	74 (1·2)
Maternal mood disorder during pregnancy, n (%)						
No	914,325 (99·9)	5,563 (99·9)	1,003,920 (99·9)	8,227 (99·9)	1001372 (99·3)	6252 (99·4)
Yes	229 (0·0)	2 (0·0)	463 (0·0)	2 (0·0)	7426 (0·7)	35 (0·6)

Basic characteristics	Birth year 1982-1990		Birth year 1991-2000		Birth year 2001-2010	
	Total	T1D	Total	T1D	Total	T1D
Unclear	603 (0·1)	1 (0·0)	366 (0·0)	4 (0·0)	0 (0·0)	0 (0·0)
Gestational age, n (%)						
Very preterm	8,379 (0·9)	32 (0·6)	10,952 (1·1)	80 (1·0)	11,588 (1·1)	59 (0·9)
Preterm	46,193 (5·0)	329 (5·9)	48,751 (4·9)	449 (5·5)	48,059 (4·8)	386 (6·1)
Early term	178,180 (19·5)	1,192 (21·4)	184,812 (18·4)	1,718 (20·9)	196,660 (19·5)	1,367 (21·7)
Term	460,487 (50·3)	2,806 (50·4)	510,874 (50·8)	4,145 (50·3)	503,421 (49·9)	3,054 (48·6)
Postterm	218,778 (23·9)	1,190 (21·4)	247,959 (24·7)	1,835 (22·3)	248,531 (24·6)	1,417 (22·5)
Unknown	3,140 (0·3)	17 (0·3)	1,401 (0·1)	6 (0·1)	539 (0·1)	4 (0·1)
Birth weight, n (%)						
<1500g	4,870 (0·5)	16 (0·3)	6,364 (0·6)	35 (0·4)	6,832 (0·7)	32 (0·5)
1500-2499g	33,068 (3·6)	174 (3·1)	35,561 (3·5)	263 (3·2)	34,573 (3·4)	201 (3·2)
2500-2999g	104,664 (11·4)	664 (11·9)	105,899 (10·5)	842 (10·2)	108,854 (10·8)	648 (10·3)
3000-3999g	611,172 (66·8)	3,685 (66·2)	659,659 (65·7)	5,325 (64·7)	664,653 (65·9)	4,112 (65·4)
≥4000g	157,193 (17·2)	1,007 (18·1)	194,125 (19·3)	1,749 (21·2)	191,676 (19·0)	1,273 (20·2)
Unknown	4,190 (0·5)	20 (0·4)	3,141 (0·3)	19 (0·2)	2,210 (0·2)	21 (0·3)
Birth weight for gestational age, n (%)						
Small for gestational age	25,583 (2·8)	130 (2·3)	23,316 (2·3)	168 (2·0)	2,1487 (2·1)	109 (1·7)
Normal for gestational age	836,271 (91·4)	5,097 (91·6)	913,587 (90·9)	7,448 (90·5)	920,069 (91·2)	5,724 (91·0)
Large for gestational age	27,969 (3·1)	213 (3·8)	35,032 (3·5)	387 (4·7)	35,731 (3·5)	287 (4·6)
Unknown	25,334 (2·8)	126 (2·3)	32,814 (3·3)	230 (2·8)	31,511 (3·1)	167 (2·7)
Birth order, n (%)						
Firt-born	375,847 (41·1)	2,290 (41·1)	407,004 (40·5)	3,336 (40·5)	445,481 (44·2)	2,705 (43·0)
Second-born	323,890 (35·4)	1,936 (34·8)	367,598 (36·6)	3,028 (36·8)	366,533 (36·3)	2,342 (37·3)
Third-born or higher	215,420 (23·5)	1,340 (24·1)	230,147 (22·9)	1,869 (22·7)	196,784 (19·5)	1,240 (19·7)
Infection during the first year of life, n (%)						
No	824,791 (90·1)	5,028 (90·3)	901,780 (89·8)	7,417 (90·1)	698,341 (69·2)	4,301 (68·4)
Yes	83,623 (9·1)	496 (8·9)	100,441 (10·0)	798 (9·7)	305,763 (30·3)	1,954 (31·1)
Unclear	6,743 (0·7)	42 (0·8)	2,528 (0·3)	18 (0·2)	4,694 (0·5)	32 (0·5)
Serious life events, n (%)						
No death in first/second-degree relatives	311,442 (34·0)	3,278 (58·9)	437,387 (43·5)	5,463 (66·4)	744,415 (73·8)	4,913 (78·1)
Death in second-degree relatives	562,968 (61·5)	2,164 (38·9)	532,400 (53·0)	2,632 (32·0)	252,454 (25·0)	1,313 (20·9)

Basic characteristics	Birth year 1982-1990		Birth year 1991-2000		Birth year 2001-2010	
	Total	T1D	Total	T1D	Total	T1D
Death in first-degree relatives	40,747 (4·5)	124 (2·2)	34,962 (3·5)	138 (1·7)	11,929 (1·2)	61 (1·0)
T1D in parents, n (%)						
No	903,744 (98·8)	5,296 (95·1)	995,376 (99·1)	7,864 (95·5)	1,001,462 (99·3)	6,033 (96·0)
Yes	3,735 (0·4)	135 (2·4)	4,677 (0·5)	226 (2·7)	4,776 (0·5)	201 (3·2)
Unclear¶	7,678 (0·8)	135 (2·4)	4,696 (0·5)	143 (1·7)	2,560 (0·3)	53 (0·8)

T1D: type 1 diabetes; BMI: body mass index; SD: standard error.

* Children were not be able to follow-up until they turned age 19 years due to the end of follow-up in April of 2020.

¶ Parental T1D status is unclear because of contradictory types of diabetes diagnosis in registers.

Supplementary Table 2. Number of concordant and discordant pairs of full siblings for type 1 diabetes overall and at age 0-6, 7-12, and 13-18 years

Age at T1D diagnosis	Concordant pairs without T1D	Discordant pairs	Concordant pairs with T1D	Total
**Overall (0-18 years)**	1,528,499	20,017	841	1,549,357
**0-6 years**	1,543,342	5,869	146	1,549,357
**7-12 years**	1,506,093	8,715	150	1,514,958
**13-18 years**	1,268,969	5,626	65	1,274,660

T1D: type 1 diabetes

Supplementary Table 3. Comparisons between models with and without moderation of birth year* on different components of type 1 diabetes variance based on all possible sibling pairs

Models	β1	β2	-2LL	*p* value¶	AIC
Overall (0-18 years)					
Model 1	1·55	0·70	255668·6		255680·6
Model 2	0·08	--	255807·3	5·19E-32	255817·3
Model 3	--	0·01	255809·3	1·86E-32	255819·3
Model 4	--	--	255809·4	2·69E-31	255817·4
Age 0-6 years					
Model 1	1·35	0·26	88003·5		88015·5
Model 2	0·57	--	88014·9	7·20E-04	88024·9
Model 3	--	0·03	88025·7	2·40E-06	88035·7
Model 4	--	--	88025·9	1·32E-05	88033·9
Age 7-12 years					
Model 1	1·43	1·16	122228·0		122240·0
Model 2	0·11	--	122335·1	4·19E-25	122345·1
Model 3	--	0·04	122337·7	1·12E-25	122347·7
Model 4	--	--	122338·5	1·02E-24	122346·5
Age 13-18 years					
Model 1	-1·46	-1·22	80940·6		80952·6
Model 2	-0·07	--	81139·5	3·51E-45	81149·5
Model 3	--	-0·06	81139·7	3·18E-45	81149·7
Model 4	--	--	81140·9	3·11E-44	81148·9

* The birth year variable in the moderation models were transformed using the formula of (birth year-1996)/28.

¶ Two-sided *p* values for likelihood ratio tests comparing models 2-4 to model 1. No adjustment for multiple comparisons was made, since this is the standard way of comparing different heritability models and this is pre-specified analysis instead of data-driven analysis.

Model 1 was adjusted for sex and birth year for the liability threshold and included the moderation of birth year on both the genetic (β_1) and non-shared environment (β_2) components to type 1 diabetes variance.

Model 2 was adjusted for sex and birth year for the liability threshold and included the moderation of birth year on the genetic component (β_1) to type 1 diabetes variance.

Model 3 was adjusted for sex and birth year for the liability threshold and included the moderation of birth year on the non-shared environment component (β_2) to type 1 diabetes variance.

Model 4 was adjusted for sex and birth year for the liability threshold, and without moderation of birth year

Supplementary Table 4. Sensitivity analysis comparing models with and without moderation of birth year* on different components of type 1 diabetes variance based on one sibling pair from each family

Models	β1	β2	-2LL	*p* value¶	AIC
Overall (0-18 years)					
Model 1	1·58	0·74	146234·1		146246·1
Model 2	0·10	--	146322·9	4·28E-21	146332·9
Model 3	--	-0·003	146324·9	1·55E-21	146334·9
Model 4	--	--	146324·9	1·86E-20	146332·9
Age 0-6 years					
Model 1	1·41	0·36	51704·0		51716·0
Model 2	0·64	--	51715·3	7·89E-04	51725·3
Model 3	--	0·02	51721·8	2·46E-05	51731·8
Model 4	--	--	51721·9	1·32E-04	51729·9
Age 7-12 years					
Model 1	1·29	0·89	70708·5		70720·5
Model 2	0·10	--	70763·1	1·47E-13	70773·1
Model 3	--	0·03	70764·4	7·80E-14	70774·4
Model 4	--	--	70764·8	6·01E-13	70772·8
Age 13-18 years					
Model 1	-1·94	-1·57	46965·4		46977·4
Model 2	-0·07	--	47080·5	7·59E-27	47090·5
Model 3	--	-0·06	47080·5	7·73E-27	47090·5
Model 4	--	--	47081·3	6·83E-26	47089·3

T1D: type 1 diabetes.

* The birth year variable in the moderation models were transformed using the formula of (birth year-1996)/28.

¶ Two-sided *p* values for likelihood ratio tests comparing models 2-4 to model 1. No adjustment for multiple comparisons was made, since this is the standard way of comparing different heritability models and this is pre-specified analysis instead of data-driven analysis.

Model 1 was adjusted for sex and birth year for the liability threshold and included the moderation of birth year on both the genetic (β_1) and non-shared environment (β_2) components to type 1 diabetes variance.

Model 2 was adjusted for sex and birth year for the liability threshold and included the moderation of birth year on the genetic component (β_1) to type 1 diabetes variance.

Model 3 was adjusted for sex and birth year for the liability threshold and included the moderation of birth year on the non-shared environment component (β_2) to type 1 diabetes variance.

Model 4 was adjusted for sex and birth year for the liability threshold, and without moderation of birth year.

Supplementary Table 5. The difference in heritability between different birth years

Birth years for comparion	Heritability difference (95% CI*)			
	**Overall (0-18 years)**	**0-6 years**	**7-12 years**	**13-18 years**
2000 vs 1985	0·010 (-0·010, 0·037)	0·181 (-0·145, 0·232)	-0·118 (-0·147, 0·142)	0
2000 vs 1990	0·003 (-0·004, 0·012)	0·073 (-0·074, 0·093)	-0·037 (-0·049, 0·044)	0
2000 vs 1995	0·001 (-0·001, 0·004)	0·025 (-0·029, 0·033)	-0·012 (-0·016, 0·014)	0
2010 vs 1985	0·011 (-0·012, 0·041)	0·207 (-0·179, 0·264)	-0·130 (-0·162, 0·156)	0
2010 vs 1990	0·004 (-0·005, 0·016)	0·099 (-0·109, 0·126)	-0·049 (-0·066, 0·058)	0
2010 vs 1995	0·002 (-0·003, 0·008)	0·051 (-0·066, 0·066)	-0·024 (-0·033, 0·029)	0

Estimates were obtained from best fitting AE models (with moderation of birth year on both genetic and non-shared environmental components) for T1D at age 0-18, 0-6 and 7-12 years. Point estimates for T1D at age 13-18 years from different AE models were similar and therefore we presented results from the AE model without any moderation to avoid model fitting problems when the data are sparse (small number of concordant pairs).

* 95% CIs were calculated using bootstrapping.

Supplementary Table 6. Basic characteristics and heritability of type 1 diabetes (0-18 years) in the simulated cohort as compared to the original cohort

Cohort	No. of T1D cases	Cumulative incidence, cases per 10000 children	Concordant pairs without T1D	Discordant pairs	Concordant pairs with T1D	Heritability (95% CI)
Original cohort*	20,086	77	1,528,499	20,017	841	0·83 (0·79, 0·86)
Simulated cohort¶	29,816	117	1,517,165	31,266	926	0·59 (0·55, 0·61)

T1D: type 1 diabetes.

*The real-world cohort in our study.

¶ The simulated cohort has an excess cumulative incidence of 40 cases per 10,000 children (vs original cohort) caused completely by environmental factors not shared within siblings. An excess cumulative incidence of 40 cases per 10,000 children was simulated because this was the magnitude of increasing T1D incidence from birth year 1982 to 2000 in our original cohort.

Supplementary Table 7. Environmental factors associated with type 1 diabetes identified through the literature review

Categories	Factors	References
Factors previously associated with T1D*	Maternal age at delivery	2010, Cardwell CR, et al;[3] 1999, Dahlquist GG, et al;[4] 2005, Cardwell CR, et al;[5] 1992, Dahlquist G, et al.[6]
	Maternal BMI/overweight/obesity during pregnancy	2019, Hidayat K, et al[7]
	Maternal smoking during pregnancy	2022, Edstorp J, et al;[8] 2022. Wei Y, et al.[9]
	Maternal infection during pregnancy	2018, Yue Y, et al;[10] 2019, Waernbaum I, et al.[11]
	Maternal educational level	2023, White PA, et al.[12]
	Maternal marital status at childbirth	2010, D'Angeli MA, et al;[13] 2008, Waldhoer T, et al.[14]
	Maternal mood disorders during pregnancy	2023, Smew AI, et al.[15]
	Maternal eating disorders and obsessive–compulsive disorder	2022, Yin W, et al.[16]
	Caesarean Section	2019, Tanoey J, et al.[17]
	Gestational age	2015, Khashan AS, et al.[18]
	Large for gestational age, small for gestational age	2023, Zhang Y, et al;[19] 2015, Khashan AS, et al.[18]
	Birth weight	2009, Harder T, et al;[20] 2010, Cardwell CR, et al;[21] 2015, Khashan AS, et al.[18]
	Birth order	2011, Cardwell CR, et al.[22]
	Born after assisted reproductive technology	2020, Norrman E, et al.[23]
	Longer duration of breastfeeding, gluten delay; exclusive breastfeeding (≥2-3 months vs <2-3 months), later introduction of cow's milk and fruit	2021, Lampousi AM, et al.[24]
	Increased early growth	2002, EURODIAB Substudy 2 Study Group;[25] 2015, Magnus MC, et al.[26]
	Childhood obesity/adiposity	2011, Verbeeten KC, et al;[1] 2017, Censin JC, et al.[27] 2022, Richardson TG, et al.[28]
	Enterovirus infection	2023, Isaacs SR, et al.[29]
	Bacterial infection	2022, Kordonouri O, et al.[30]
	Antibiotic treatment	2022, Duong QA, et al;[31] 2020, Wernroth ML, et al.[32]
	Serious life events such as death in family members or separation in parents	2016, Rewers M, et al.[33]
Factors associated with T1D also in Mendelian randomization studies†	Childhood obesity/adiposity	2017, Censin JC, et al;[27] 2022, Richardson TG, et al.[28]

Factors associated with T1D also in sibling comparison studies†	Gestational age	2015, Khashan AS, et al.[18]
	Small for gestational age (vs appropriate for gestational age)	2015, Khashan AS, et al.[18]
	Birth weight<1500g (vs birth weight of 3000–3999g)	2015, Khashan AS, et al.[18]
	Maternal smoking during pregnancy	2022, Wei Y, et al.[9]
	Maternal mood disorders during pregnancy (the association was observed only in children surviving without T1D after age 8 years)	2023, Smew AI, et al.[15]
	Early childhood antibiotic treatment	2020, Wernroth ML, et al.[32]
Factors inappropriate for sibling comparison analysis¶	Maternal age at delivery	
	Maternal educational level	
	Serious life events	
	Birth order	
Factors not associated with T1D in previous sibling comparison studies	Mode of delivery	2014, Khashan AS, et al.[34]
	Large for birth weight (vs appropriate for gestational age)	2015, Khashan AS, et al.[18]
	birth weight of 1500–2499, 2500-2999g, or 4000-5500g (vs birth weight of 3000–3999g)	2015, Khashan AS, et al.[18]

T1D: type 1 diabetes; BMI: body mass index.

* These factors (except for separation in parents, antibiotic treatment, dietary factors, being born after assisted reproductive technology, which are unavailable in our data) were explored in the current study to investigate the association with T1D in Swedish registers, supplemented with sibling comparison design when possible.

† These factors are regarded as potential factors that causally affect T1D in the Swedish population.

¶ Reasons why these factors are inappropriate for sibling analysis: within each sibling group, as compared to siblings born earlier, those born later always have a higher maternal age at delivery, a higher birth order, and a higher (or at least not lower) maternal educational level, and they also have less (or at least not more) experience of serious life events (deaths in relatives after birth).

Supplementary Table 8. ICD codes for infection

	ICD-10	ICD-9	ICD-8
Any infection	A00-A99; B00-B89; B95-B99; E06.0; G00-G02; G04; G05-G08; H00; H03; H10; H66; H70; I00-I02; I30; I33; J00-J06; J09; J10-J18; J20-J22; J32; J36; J85; J86; K35-K37; K57.0; K57.2; K57.4; K57.8; K61; K63.0; K67; K75.0; K77.0; K81.0; K83.0; K85; L00-L04; L08; M00; M01; M46.3; M86; N10-N12; N30; N61; N70-N76; O07.0; O07.5; O08.0; O23; O35.3; O41.1; O75.3; O98; P23*; P35-39*	001-018; 020-027; 030-041; 045-057; 060-066; 070-088; 090-104; 110-112; 114-118; 120-136; 245A; 254B; 320; 321; 323; 324; 372A; 372C; 382; 383; 390; 391; 392; 420; 421; 460-466; 473; 475; 480-487; 510; 513; 540-542; 566; 572A; 575A; 590; 595; 614-616; 646F; 646G; 647; 658E; 659C; 659D; 680-684; 686; 730; 770A*; 771*	000-018; 020-027; 030-043; 045; 046; 050-057; 060-065; 067; 068; 070-076; 078-104; 110-117; 120-134; 136; 320-322; 360,00; 360,01; 361; 362; 381-383; 390-392; 420-422; 460-466; 470-474; 480-486; 490; 501; 503; 510; 513; 540; 541; 562,02; 562,11; 566; 567; 569,00; 569,01; 569,02; 569,03; 569,04; 577,01; 590; 595; 597; 612-614; 616; 620; 622; 630; 635; 636,00; 670-672; 678; 680-684; 686; 710; 720; 732; 761,00; 761,20; 761,30; 761,40; 761,80; 763,00; 999,30
ICD codes to be treated as a separate group†	E32; H01; H04; H05; H13; H16; H32; H44; H60; H62; I40; J35; J39; K04; K05; K11; K12; K14; K65; L05; L30; M49; M60; M63; M65; N13; N15; N34; N39; N98; T80-T84; T88	279; 360; 370; 373; 375; 376; 380; 422; 478; 511; 522; 523; 527-529; 562; 567; 597; 599; 611; 685; 711; 728; 790; 996; 998; 999	366-369; 380; 384; 508; 511; 522; 527; 528; 542; 599; 611; 629; 789; 998
Virus infection	A08; A60; A80-A89; A92-A99; B00-B09; B15-B19; B20-B34; B97; G02.0; G05.1; J00; J09-J12; O35.3; O98.4-98.5; P23.0*; P35*	045-066; 070-072; 074-075; 077; 079; 321E, H; 323A, C, D; 460; 647F, G; 771A, B*	040-043; 045-065; 067-072; 074; 075; 078; 460; 470-474; 480; 761,20; 761,30
Bacterial infection	A00-A05; A15-A19; A20-A28; A65-A79; B95-B96; E06.0; G00; G01; G04.2; G05.0; I00-I02; J13-J15; J85; K57.0; K57.2; K57.4; K57.8; K61; K63.0; K81.0; K85; L00-L04; M00; N61; N74; O07.0; O07.5; O23; O41.1; O75.3; O98.0-98.2; P23.1-23.6*; P36*; P37.0*;	001-005; 010-041; 073; 076; 080-083; 087-098; 100-104; 245A; 320; 390-392; 481; 482; 513; 566; 646F,G; 647A, B, D; 658E; 659D; 681-684; 771D*	000-005; 010-018; 020-039; 073; 076; 080-083; 088-104; 320,00-320,80; 361; 390-392; 481; 482; 513; 562,02; 562,11; 566; 569,00; 577,01; 630; 635; 636,00; 678,02; 680-684; 710; 761,00; 763,00; 999,30

	ICD-10	ICD-9	ICD-8
Genitourinary infection	A50-A64; O23; N10-N12; N30; N70-N76; P39.3*	016; 090-099; 590; 595; 614-616; 646G	016; 090-099; 590; 595; 612-614; 620; 622; 635
Respiratory infection	A15-A16; A37-A38; B27; B39-42; B44; B59; J00-J06; J09-J18; J20-J22; J32; J36; J85; J86; P23*	010-012; 033-034; 460-466; 473; 475; 480-487; 510; 513; 770A*	010-012; 460-466; 470-473; 480-486; 501; 503; 510; 513
Gastrointestinal Infection	A00-A09	001-009	000-009

* only for childhood infection

† For several ICD codes that we don’t have the necessary level of detail (4 or 5 positions), we categorize individuals with the corresponding ICD codes at 3 positions as a separate group since we were unsure about their infection status.

Supplementary Method 1. Assessment of childhood-onset type 1 diabetes

Participants were followed-up for T1D diagnosis in the National Patient Register (NPR) and National Diabetes Register (NDR) from birth until they turned 19 years, age of emigration or death (through the Total Population Register), or age in 2020, whichever came first. NPR was established in 1964 and covers almost all inpatient records in Sweden since 1982[35] and records outpatient care since 2001.[36] NDR was established in 1996 and gathers data on both incident and prevalent diabetes cases from Swedish health centers since then, encompassing 90% of the total patient population. The positive predictive value of a T1D diagnosis is 0·97 in NDR and 0.95 in NPR for people age ≤30 years.[37, 38] Self-reported year at diabetes diagnosis was available for most cases in NDR, even for those diagnosed before 1996. Childhood-onset T1D was defined as a T1D diagnosis recorded in NDR or NPR (ICD-8/ICD-9 code 250, ICD-10 code E10) before age 19 years. For cases initially recorded with the ICD-8/ICD-9 code of 250 in NPR (n=2930), a later record of E10 (ICD-10 code) in NPR or a T1D record in NDR was required to establish T1D diagnosis (n=2819, 96.2% of all cases initially recorded with ICD-8/ICD-9 250). The date of diagnosis was the earliest recorded visit to NPR or NDR, or December 31st of the self-reported year at diagnosis in NDR, or the date of the first prescription for glucose-lowering drugs in the Swedish National Prescribed Drug Register, whichever came first.

Supplementary Method 2. PubMed search terms to identify type 1 diabetes-related environmental factors

(environment OR prenatal OR perinatal OR maternal OR environmental OR “early life” OR childhood OR obesity OR diet OR infection OR antibiotic OR breastfeeding OR diet OR food) AND ("Diabetes Mellitus, Type 1"[Mesh] OR “type 1 diabetes” OR “T1DM” OR “T1D”)

Supplementary Method 3. Assessment of environmental factors

The Medical Birth Register (MBR) provided information on birth year and month, sex, gestational age (categorized as very preterm (22–32 weeks), preterm (33-36 weeks), early term (37-38 weeks), term (39-40 weeks) and postterm (≥41 weeks)[18]), birth weight (categorized into <1500g, 1500-2499g, 2500-2999g, 3000-3999g, and ≥4000g), birth weight for gestational age (small for gestational age [below mean - 2 SD],[39] normal, and large for gestational age [above mean +2 SD][40]), mode of delivery, maternal marital (cohabiting) status during pregnancy, maternal age at delivery, maternal smoking and body mass index (BMI) during pregnancy. Information on parental educational attainment was retrieved from the Longitudinal Integrated Database for Health Insurance and Labor Market Studies[41]. Exposure to serious life events was defined as deaths in first- and second-degree relatives after the participants' birth. Diagnoses of maternal mood disorder during pregnancy, maternal infections during pregnancy and infections during the first year of life were retrieved from the National Patient Register (NPR). Maternal infection during pregnancy was defined as any infection recorded from the month of conception to the month of childbirth. The ICD codes for any infection,[42, 43] types of infection,[42] and sites of infection[42, 44] (Supplementary Table 8) were adopted from previous studies.

Supplementary Method 4. Fitting liability threshold model with moderation effects by birth year

To explore the potential changes in the relative contributions of genetic (A) and environmental factors (E) over birth year, we fitted liability threshold models[45] with moderation effects of birth year on the variance of genetic component (model 2), environmental component (model 3), or both (model 1), with adjustment for sex and birth year for the liability threshold. The moderation modelling was performed by incorporating linear regressions on path coefficients, which partitioned the corresponding component of variance into a part unrelated to the moderator and a part associated with the moderator[46, 47] (**Supplementary Figure 2**). We fixed the total T1D (type 1 diabetes) variance at 1 at the median value of the birth year (1996) and allowed the total variance to change over birth years. We then compared model 1, 2, 3 and the model without any moderation effect (model 4) based on likelihood ratio test (-2LL) and Akaike information criterion (AIC) values. Note, though, that we only interpret the proportions of variance explained by A and E, since it is not possible to infer the absolute variance with only binary outcome variables. Sibling pairs from the same family are not independent of each other. Not accounting for such dependence between sibling pairs may lead to favoring more complex models (models 1-3 vs 4). Therefore, to ensure selecting the best fitting model, we performed a sensitivity analysis by comparing -2LL and AIC between models 1-4 based on only one full sibling pair from each family. The best fitting model was used to estimate the heritability and the proportion of non-shared environmental contribution in each birth year as the main results. The 95% CIs for heritability and non-shared environment contribution were estimated using bootstrapping to account for dependency between sibling pairs. Bootstrapping was performed by randomly drawing as many families (with replacement) as in the original dataset 1000 times, and the middle 95% (2·5% to 97·5%) of the produced distribution of parameters were used as CIs.[48]

We also estimated the heritability in each birth year for T1D at age 0-6, 7-12, and 13-18 years, separately. The calculation of heritability for T1D at age 7-12 years were based on children who were still alive and free of T1D before age 7 years and that for T1D at age 13-18 years was based on children who were still alive and free of T1D before age 13 years. The best fitting model among models 1-4 was used for the calculation of heritability, except for T1D at age 13-18 years, for which the point estimates were similar across models 1-4 and results from the simplest model (model 4) were presented to avoid model fitting problems when the sample size is limited.

Supplementary Method 5. Simulation analysis to estimate expected heritability in a scenario where the increasing incidence of type 1 diabetes is completely driven by environmental factors

If the increasing incidence of type 1 diabetes (T1D) over time is mainly driven by changes in environmental factors, the relative contribution of environmental factors to T1D variance would be expected to increase and heritability decrease over time.[49] We performed a simulation analysis to estimate the expected heritability of T1D (age 0-18 years) in the scenario where an increasing cumulative incidence of 40 cases per 10,000 children over time is completely driven by changes in environmental factors. To establish the simulated cohort, we made a copy of our original cohort of participants born in 1982-2010 and randomly assigned 0·4% of those originally censored at the end of follow-up to have T1D occurrence in the simulated cohort. Therefore, the cumulative incidence of T1D in the simulated cohort was increased by 40 cases per 10,000 children compared to the original cohort. The random assignment process means that the increasing incidence in participants was independent of the increasing incidence in their siblings. Therefore, the random assignment simulated the scenario where the increasing incidence is completely due to environmental factors not shared within siblings. We then fitted a liability threshold model (AE model) to estimate the heritability based on all possible full sibling pairs in the simulated cohort.

Supplementary Method 6. Analysis of the association between environmental factors and type 1 diabetes

We comprehensively assessed the associations of all potential and available type 1 diabetes (T1D)-related environmental factors, with T1D in the full cohort. Cox models were fitted to estimate the HR (95% CI) of T1D in relation to these exposures, with attained age as the time scale, with adjustment for birth year, sex, maternal age at delivery, maternal BMI and smoking during pregnancy, and parental country of birth and history of T1D when appropriate, and with cluster-robust standard errors. In addition, we used a sibling comparison design to reduce confounding bias with Cox models stratified by sibling groups. Such a design compares outcome hazards among siblings with different exposure status and can control for unmeasured confounding factors (genetic and environmental) shared within families.[42] An HR close to 1 in the sibling analysis indicates that an association observed in the overall cohort is due to confounding factors shared within families. The sibling comparison analysis was inappropriate and therefore not performed for maternal age at delivery, maternal educational level around birth, birth order and serious life events. The reason is that within siblings born by the same mother, those born later will have outcomes at a later calendar time and will always have a higher maternal age at delivery and birth order, may have higher (or at least not lower) maternal educational level, and are less likely to have experienced a serious life event. We also performed a negative control analysis by analyzing the association between maternal pre-pregnancy infection (infection within 1 year prior to pregnancy) and T1D in the full cohort, to detect potential bias in the analysis of maternal infection during pregnancy. Participants with missing values on categorical covariates were treated as a separate group in the analyses, and those with missing values on continuous covariates were assigned the median value with a binary variable included in the analyses to indicate if values were imputed. The follow-up duration in all Cox models was calculated

from birth, except for the exposure of own infection, which was calculated after 15 months of birth, to minimize reverse causation[32].

Supplementary Method 7. Estimating proportion of increasing incidence of type 1 diabetes (birth year 2000 vs 1982) explained by the changing prevalence of childhood overweight/obesity

We lacked individual-level data for childhood overweight/obesity and estimated the proportion of increasing (cumulative) incidence of type 1 diabetes (T1D) in birth year 2000 versus 1982 explained by the changing prevalence of childhood overweight/obesity using the following formulas.

(1) Population attributable risk[50] due to childhood overweight/obesity in 2000= $I_0\times(RR_{overweight} -1)\times P^{2000}_{overweight} + I_0\times(RR_{obese} -1)\times P^{2000}_{obese}$

I_0 is the cumulative incidence of T1D before age 19 years in a population without anyone having any risk factor for T1D. RR_{obese} and $RR_{overweight}$ are the relative risks of T1D in relation to childhood overweight and childhood obesity, respectively. $P^{2000}_{overweight}$ and P^{2000}_{obese} are the prevalence of childhood overweight and obesity for individuals born in 2000.

(2) Population attributable risk due to childhood overweight/obesity in 1982= $I_0\times(RR_{overweight} -1)\times P^{1982}_{overweight} + I_0\times(RR_{obese} -1)\times P^{1982}_{obese}$

$P^{1982}_{overweight}$ and P^{1982}_{obese} are the prevalence of childhood overweight and obesity for individuals born in 1982.

(3) Increasing cumulative incidence of T1D attributed to changing prevalence of childhood overweight/obesity from 1982 to 2000=(1)-(2)= $I_0\times [(RR_{overweight} -1)\times(P^{2000}_{overweight} - P^{1982}_{overweight})+ (RR_{obsese} -1)\times(P^{2000}_{obese} - P^{1982}_{obese})]$

(4) Total increasing cumulative incidence of T1D for birth year 2000 vs 1982=$I_{2000} - I_{1982}$

I_{2000} and I_{1982} are the cumulative incidence of T1D before age 19 years for participants born in 2000 and 1982, respectively.

(5) Proportion of increasing cumulative incidence of T1D in 2000 vs 1982 explained by changing prevalence of childhood overweight/obesity = (4)/(5) = $\mathbf{I_0\times[(RR_{overweight} -1)\times(P^{2000}_{overweight} - P^{1982}_{overweight})+ (RR_{obsese} -1)\times(P^{2000}_{obese} - P^{1982}_{obese})]/(I_{2000}-I_{1982})}$

RR_{obese} and $RR_{overweight}$ were obtained from a previous meta-analysis[1] while $P^{1982}_{overweight}$, P^{1982}_{obese}, $P^{2000}_{overweight}$, and P^{2000}_{obese} were obtained from a previous study with Swedish nationwide prevalence data[2].

I_{2000} and I_{1982} were obtained from individual-level data from our study participants. **I_0 is unknown and needs to be estimated.**

The formula that uses population attributable risk to estimate the proportion of increasing T1D incidence explained by changing prevalence of environmental factors also applies to other T1D-related factors including maternal smoking during pregnancy (Pro_{smok}), for which the proportion (**Pro_{smok}**) has been **estimated with individual-level data using causal mediation model**[51]. Once Pro_{smok} is obtained from the causal mediation model, I_0 can be estimated using the following formula (6) which uses population attributable risk to estimate Pro_{smok}:

(6) $Pro_{smok} = I_0 \times (RR_{nonsmok} - 1) \times (P^{2000}_{nonsmok} - P^{1983}_{nonsmok}) / (I_{2000} - I_{1983})$

$RR_{nonsmok}$: the relative risk of T1D in relation to maternal non-smoking during pregnancy. It was estimated as the hazard ratio of T1D in relation to maternal non-smoking during pregnancy using individual data in our study participants.

$P^{2000}_{nonsmok}$ and $P^{1983}_{nonsmok}$: prevalence of maternal non-smoking during pregnancy in our participants in the birth year of 2000 and 1983, respectively.

I_{2000} and I_{1983}: total cumulative incidence of T1D before age 19 years for our study participants born in 2000 and 1983, respectively.

In formula (6), we used 1983 instead of 1982 as the reference year due to the high missing rate of maternal smoking during pregnancy in the Medical Birth Register in 1982.

(7) **Once the value of I_0 is derived from formula (6), the proportion of interest can be estimated using formula (5).**

Supplementary References

1. Verbeeten KC, Elks CE, Daneman D, Ong KK. Association between childhood obesity and subsequent Type 1 diabetes: a systematic review and meta-analysis. *Diabet Med* **28**, 10-18 (2011).

2. NCD Risk Factor Collaboration (NCD-RisC). Worldwide trends in underweight and obesity from 1990 to 2022: a pooled analysis of 3663 population-representative studies with 222 million children, adolescents, and adults. *Lancet* **403**, 1027-1050 (2024).

3. Cardwell CR*, et al.* Maternal age at birth and childhood type 1 diabetes: a pooled analysis of 30 observational studies. *Diabetes* **59**, 486-494 (2010).

4. Dahlquist GG, Patterson C, Soltesz G. Perinatal risk factors for childhood type 1 diabetes in Europe. The EURODIAB Substudy 2 Study Group. *Diabetes Care* **22**, 1698-1702 (1999).

5. Cardwell CR, Carson DJ, Patterson CC. Parental age at delivery, birth order, birth weight and gestational age are associated with the risk of childhood Type 1 diabetes: a UK regional retrospective cohort study. *Diabet Med* **22**, 200-206 (2005).

6. Dahlquist G, Källén B. Maternal-child blood group incompatibility and other perinatal events increase the risk for early-onset type 1 (insulin-dependent) diabetes mellitus. *Diabetologia* **35**, 671-675 (1992).

7. Hidayat K, Zou SY, Shi BM. The influence of maternal body mass index, maternal diabetes mellitus, and maternal smoking during pregnancy on the risk of childhood-onset type 1 diabetes mellitus in the offspring: Systematic review and meta-analysis of observational studies. *Obes Rev* **20**, 1106-1120 (2019).

8. Edstorp J, Lampousi AM, Carlsson S. Parental smoking, type 1 diabetes, and islet autoantibody positivity in the offspring: A systematic review and meta-analysis. *Diabet Med* **39**, e14830 (2022).

9. Wei Y*, et al.* Maternal smoking during pregnancy and type 1 diabetes in the offspring: a nationwide register-based study with family-based designs. *BMC Med* **20**, 240 (2022).

10. Yue Y*, et al.* Maternal infection during pregnancy and type 1 diabetes mellitus in offspring: a systematic review and meta-analysis. *Epidemiol Infect* **146**, 2131-2138 (2018).

11. Waernbaum I, Dahlquist G, Lind T. Perinatal risk factors for type 1 diabetes revisited: a population-based register study. *Diabetologia* **62**, 1173-1184 (2019).

12. White PA, Faresjö T, Jones MP, Ludvigsson J. Low maternal education increases the risk of Type 1 Diabetes, but not other autoimmune diseases: a mediating role of childhood BMI and exposure to serious life events. *Sci Rep* **13**, 6166 (2023).

13. D'Angeli MA, Merzon E, Valbuena LF, Tirschwell D, Paris CA, Mueller BA. Environmental factors associated with childhood-onset type 1 diabetes mellitus: an exploration of the hygiene and overload hypotheses. *Arch Pediatr Adolesc Med* **164**, 732-738 (2010).

14. Waldhoer T, Rami B, Schober E. Perinatal risk factors for early childhood onset type 1 diabetes in Austria - a population-based study (1989-2005). *Pediatr Diabetes* **9**, 178-181 (2008).

15. Smew AI*, et al.* Maternal depression or anxiety during pregnancy and offspring type 1 diabetes: a population-based family-design cohort study. *BMJ Open Diabetes Res Care* **11**, (2023).

16. Yin W, Persson M, Sandin S. Parental history of psychiatric disorders and risk of type 1 diabetes in the offspring. *Diabetes Metab* **49**, 101392 (2023).

17. Tanoey J, Gulati A, Patterson C, Becher H. Risk of Type 1 Diabetes in the Offspring Born through Elective or Non-elective Caesarean Section in Comparison to Vaginal Delivery: a Meta-Analysis of Observational Studies. *Curr Diab Rep* **19**, 124 (2019).

18. Khashan AS*, et al.* Gestational Age and Birth Weight and the Risk of Childhood Type 1 Diabetes: A Population-Based Cohort and Sibling Design Study. *Diabetes Care* **38**, 2308-2315 (2015).

19. Zhang Y, Liu P, Zhou W, Hu J, Cui L, Chen ZJ. Association of large for gestational age with cardiovascular metabolic risks: a systematic review and meta-analysis. *Obesity (Silver Spring)* **31**, 1255-1269 (2023).

20. Harder T, Roepke K, Diller N, Stechling Y, Dudenhausen JW, Plagemann A. Birth weight, early weight gain, and subsequent risk of type 1 diabetes: systematic review and meta-analysis. *Am J Epidemiol* **169**, 1428-1436 (2009).

21. Cardwell CR*, et al.* Birthweight and the risk of childhood-onset type 1 diabetes: a meta-analysis of observational studies using individual patient data. *Diabetologia* **53**, 641-651 (2010).

22. Cardwell CR*, et al.* Birth order and childhood type 1 diabetes risk: a pooled analysis of 31 observational studies. *Int J Epidemiol* **40**, 363-374 (2011).

23. Norrman E*, et al.* Type 1 diabetes in children born after assisted reproductive technology: a register-based national cohort study. *Hum Reprod* **35**, 221-231 (2020).

24. Lampousi AM, Carlsson S, Löfvenborg JE. Dietary factors and risk of islet autoimmunity and type 1 diabetes: a systematic review and meta-analysis. *EBioMedicine* **72**, 103633 (2021).

25. Rapid early growth is associated with increased risk of childhood type 1 diabetes in various European populations. *Diabetes Care* **25**, 1755-1760 (2002).

26. Magnus MC*, et al.* Infant Growth and Risk of Childhood-Onset Type 1 Diabetes in Children From 2 Scandinavian Birth Cohorts. *JAMA Pediatr* **169**, e153759 (2015).

27. Censin JC, Nowak C, Cooper N, Bergsten P, Todd JA, Fall T. Childhood adiposity and risk of type 1 diabetes: A Mendelian randomization study. *PLoS Med* **14**, e1002362 (2017).

28. Richardson TG*, et al.* Childhood body size directly increases type 1 diabetes risk based on a lifecourse Mendelian randomization approach. *Nat Commun* **13**, 2337 (2022).

29. Isaacs SR*, et al.* Enteroviruses and risk of islet autoimmunity or type 1 diabetes: systematic review and meta-analysis of controlled observational studies detecting viral nucleic acids and proteins. *Lancet Diabetes Endocrinol*, (2023).

30. Kordonouri O*, et al.* Infections in the first year of life and development of beta cell autoimmunity and clinical type 1 diabetes in high-risk individuals: the TRIGR cohort. *Diabetologia* **65**, 2098-2107 (2022).

31. Duong QA, Pittet LF, Curtis N, Zimmermann P. Antibiotic exposure and adverse long-term health outcomes in children: A systematic review and meta-analysis. *J Infect* **85**, 213-300 (2022).

32. Wernroth ML*, et al.* Early Childhood Antibiotic Treatment for Otitis Media and Other Respiratory Tract Infections Is Associated With Risk of Type 1 Diabetes: A Nationwide Register-Based Study With Sibling Analysis. *Diabetes Care* **43**, 991-999 (2020).

33. Rewers M, Ludvigsson J. Environmental risk factors for type 1 diabetes. *The Lancet* **387**, 2340-2348 (2016).

34. Khashan AS, Kenny LC, Lundholm C, Kearney PM, Gong T, Almqvist C. Mode of obstetrical delivery and type 1 diabetes: a sibling design study. *Pediatrics* **134**, e806-813 (2014).

35. Socialstyrelsen. Täckningsgrad för den somatiska och psykiatriska slutenvården.).

36. Ludvigsson JF*, et al.* External review and validation of the Swedish national inpatient register. *BMC Public Health* **11**, 450 (2011).

37. Eeg-Olofsson K*, et al.* Glycemic control and cardiovascular disease in 7,454 patients with type 1 diabetes: an observational study from the Swedish National Diabetes Register (NDR). *Diabetes Care* **33**, 1640-1646 (2010).

38. Miao J, Brismar K, Nyrén O, Ugarph-Morawski A, Ye W. Elevated hip fracture risk in type 1 diabetic patients: a population-based cohort study in Sweden. *Diabetes Care* **28**, 2850-2855 (2005).

39. Marsál K, Persson PH, Larsen T, Lilja H, Selbing A, Sultan B. Intrauterine growth curves based on ultrasonically estimated foetal weights. *Acta Paediatr* **85**, 843-848 (1996).

40. Lindell G, Maršál K, Källén K. Predicting risk for large-for-gestational age neonates at term: a population-based Bayesian theorem study. *Ultrasound Obstet Gynecol* **41**, 398-405 (2013).

41. Ludvigsson JF, Svedberg P, Olén O, Bruze G, Neovius M. The longitudinal integrated database for health insurance and labour market studies (LISA) and its use in medical research. *Eur J Epidemiol* **34**, 423-437 (2019).

42. Brynge M, Sjöqvist H, Gardner RM, Lee BK, Dalman C, Karlsson H. Maternal infection during pregnancy and likelihood of autism and intellectual disability in children in Sweden: a negative control and sibling comparison cohort study. *Lancet Psychiatry* **9**, 782-791 (2022).

43. Al-Haddad BJS*, et al.* Long-term Risk of Neuropsychiatric Disease After Exposure to Infection In Utero. *JAMA Psychiatry* **76**, 594-602 (2019).

44. Khalili H, Axelrad JE, Roelstraete B, Olén O, D'Amato M, Ludvigsson JF. Gastrointestinal Infection and Risk of Microscopic Colitis: A Nationwide Case-Control Study in Sweden. *Gastroenterology* **160**, 1599-1607.e1595 (2021).

45. Skov J, Kuja-Halkola R, Magnusson PKE, Gudbjörnsdottir S, Kämpe O, Bensing S. Shared etiology of type 1 diabetes and Hashimoto's thyroiditis: a population-based twin study. *Eur J Endocrinol* **186**, 677-685 (2022).

46. Medland SE, Neale MC, Eaves LJ, Neale BM. A note on the parameterization of Purcell's G x E model for ordinal and binary data. *Behav Genet* **39**, 220-229 (2009).

47. Purcell S. Variance components models for gene-environment interaction in twin analysis. *Twin Res* **5**, 554-571 (2002).

48. Wei Y, Liu S, Andersson T, Feychting M, Kuja-Halkola R, Carlsson S. Familial aggregation and heritability of childhood-onset and adult-onset type 1 diabetes: a Swedish register-based cohort study. *Lancet Diabetes Endocrinol*, (2024).

49. Taylor MJ*, et al.* Etiology of Autism Spectrum Disorders and Autistic Traits Over Time. *JAMA Psychiatry* **77**, 936-943 (2020).

50. Gordis L. More on risk: estimating the potential for prevention. In: *Epidemiology*). Elsevier Inc (2014).

51. Ohm J*, et al.* Socioeconomic Disparities and Mediators for Recurrent Atherosclerotic Cardiovascular Disease Events After a First Myocardial Infarction. *Circulation* **148**, 256-267 (2023).
