# Supplementary material for: Stable heritability of type 1 diabetes in a Swedish Nationwide Cohort Study
Source: Nat Commun. 2025 Jun 17;16:5327. doi: 10.1038/s41467-025-60813-2 (PMC12174315; doi:10.1038/s41467-025-60813-2)
Supplement: Supplementary file 2 — Description of Additional Supplementary Files [file 41467_2025_60813_MOESM2_ESM.pdf]

Description of Additional Supplementary Files

Supplementary Data 1: Proportion of excess cumulative incidence of type 1 diabetes (birth year 2000 vs 1982) explained by changing prevalence of childhood overweight/obesity under different circumstances*
